# Autocrine regulation of cell proliferation by estrogen receptor-alpha in estrogen receptor-alpha-positive breast cancer cell lines

**DOI:** 10.1186/1471-2407-9-31

**Published:** 2009-01-26

**Authors:** Huining Tan, Yili Zhong, Zhongzong Pan

**Affiliations:** 1 Department of Animal Science, Vermont Cancer Center, University of Vermont, Burlington, VT 05405, USA

## Abstract

**Background:**

Estrogen receptor-α (ERα) is essential for mammary gland development and is a major oncogene in breast cancer. Since ERα is not colocalized with the cell proliferation marker Ki-67 in the normal mammary glands and the majority of primary breast tumors, it is generally believed that paracrine regulation is involved in ERα mediated cell proliferation. In the paracrine model, ERα-positive cells don't proliferate but will release some paracrine growth factors to stimulate the neighboring cells to proliferate. In a subpopulation of cancer cells in some primary breast tumors, however, ERα does colocalize with the cell proliferation marker Ki-67, suggesting an autocrine regulation by ERα in some primary breast tumors.

**Methods:**

Colocalization of ERα with Ki-67 in ERα-positive breast cancer cell lines (MCF-7, T47D, and ZR75-1) was evaluated by immunofluorescent staining. Cell cycle phase dependent expression of ERα was determined by co-immunofluorescent staining of ERα and the major cyclins (D, E, A, B), and by flow cytometry analysis of ERα^high ^cells. To further confirm the autocrine action of ERα, MCF-7 cells were growth arrested by ICI182780 treatment, followed by treatment with EGFR inhibitor, before estrogen stimulation and analyses for colocalization of Ki-67 and ERα and cell cycle progression.

**Results:**

Colocalization of ERα with Ki-67 was present in all three ERα-positive breast cancer cell lines. Unlike that in the normal mammary glands and the majority of primary breast tumors, ERα is highly expressed throughout the cell cycle in MCF-7 cells. Without E2 stimulation, MCF-7 cells released from ICI182780 treatment remain at G1 phase. E2 stimulation of ICI182780 treated cells, however, promotes the expression and colocalization of ERα and Ki-67 as well as the cell cycle progressing through the S and G2/M phases. Inhibition of EGFR signaling does not inhibit the autocrine action of ERα.

**Conclusion:**

Our data indicate that ERα can mediate estrogen-induced cell proliferation in an autocrine mode in ERα-positive breast cancer cell lines. All of the three ERα-positive cell lines used in our study showed colocalization of ERα and Ki-67, indicating that these cell lines might be originated from primary tumor cells with autocrine regulation.

## Background

The ovarian-derived steroid hormone estrogen is one of the main regulators of mammary epithelial cell proliferation and differentiation. While estrogen is required for normal mammary gland development, cumulative exposure to estrogen during a woman's lifetime is a high risk factor for breast malignancy. The action of estrogen is exerted by binding and activating the estrogen receptors, ERα and ERβ. Studies on the expression patterns of estrogen receptors and gene knock-out mouse models indicate that ERα is the primary estrogen receptor for mammary epithelial cell proliferation and differentiation. While ERα is expressed exclusively in the epithelial cells, ERβ is expressed in both epithelial and stromal cells but the expression of ERβ in the epithelial cells is very weak [1-4]. In ERβ knock-out mice, the mammary glands can still form the bronchoalveolar structure and lactate normally [5,6]. In ERα knock-out mice, however, the mammary gland remains at rudimentary duct stage without further development [7-9]. While the role of ERβ in breast cancer is less understood, ERα is one of the most known oncogenes in breast cancer [10-12]. Abnormal expression of ERα is found in about 70–80% of human breast cancers, and about 50% of ERα-positive breast cancer patients respond to anti-estrogen therapy [10,13]. When overexpressed in the mammary gland epithelial cells of transgenic mice, ERα leads to mammary malignancy [14,15].

Despite the significant role of ERα in mediating estrogen-induced cell proliferation in normal mammary development and breast cancer, the mechanism is still not fully understood. In the normal mammary gland, ERα expression is found only in a subpopulation of the mammary epithelial cells and the percentage of ERα-positive cells is affected by the physiologic conditions. Unlike most other cellular receptors, ERα expression is not found in proliferating mammary epithelial cells [1-3,16-18]. This observation leads to the prevailing concept that ERα mediates cell proliferation in a paracrine manner [1,19]. In this paracrine model, ERα-positive cells don't proliferate; the ERα-positive cells, however, when stimulated by estrogen, will produce and release paracrine growth factors, which in turn stimulate the neighboring cells to proliferate [1,9]. The EGF family member amphiregulin could be one of those important paracrine growth factors involved in ERα-mediated mammary gland epithelial cell proliferation [20]. It is not clear why ERα-positive cells cannot proliferate, or why ERα does not stimulate cell proliferation in an autocrine mode or in both autocrine and paracrine modes. A recent study by Cheng et al. challenged the paradigm that ERα cannot mediate ERα-positive cell proliferation [17]. The authors proposed that ERα is degraded early in the cell cycle, which accounts for the dissociation of the ERα-staining and cell proliferation marker.

In contrast to the mechanism in normal mammary tissues, ERα might mediate cancer cell proliferation via paracrine and/or autocrine modes in primary breast cancers. In ERα-positive primary breast cancers, the percentage of ERα-staining cells varies from 10% to 50% or higher [1,3,21,22]. Most of the ERα-staining cells are not stained with Ki-67 in these ERα-positive primary breast tumors [1,3,23,24]. In some primary breast tumors, however, ERα does colocalize with the cell proliferation marker Ki-67 in a sub-population of tumor cells; the percentage of ERα and Ki-67 duel-staining cells varies among different patients, from 0% to ~5% of total cells [1,3]. The co-staining of ERα and Ki-67 suggests that ERα-positive cells in these primary breast tumors might be capable of proliferating, i.e., ERα might be able to mediate cell proliferation via autocrine fashion in these primary breast tumor cells.

Using breast cancer cell line models, ERα has been shown to have non-genomic effect in addition to the traditional genomic action [25]. In its genomic action as a transcriptional factor, ERα activation in MCF-7 cells can induce the expression of cyclin D and myc to promote cell cycle progression [26-29]. When stimulated by estrogen, the effect on cell proliferation in MCF-7 cells is usually not obvious until 5–7 days after the stimulation [30-32]. It is not clear why the impact is delayed for such long time and that raises the question whether the activation of ERα alone is sufficient to drive cell cycle progression through all the phases for mitosis. For the non-genomic action of ERα, cell signaling initiated from the cell surface can activate multiple pathways such as the ERK and AKT pathways [33-35]. Using the EGFR inhibitor, Levine and colleagues demonstrated that EGFR is required for the cell surface ERα-activated signaling transduction [35]. Marks and colleagues demonstrated that inhibition of the MAPK and PI3K-AKT pathways can prevent estrogen-induced mitogenesis in MCF-7 cells [36]. Considering all of the information derived from different perspectives, it is very likely that MCF-7 cells and some other ERα-positive breast cancer cell lines might be regulated by ERα via the autocrine as well as the paracrine modes. In this study, we demonstrated that ERα is colocalized with Ki-67 in MCF-7, T47D, and ZR75-1 cells, the ERα-positive breast cancer cell lines used in our study. Using MCF-7 cell line, we demonstrated that ERα is present in all the phases of cell cycle and activation of ERα in G1 phase promotes cell cycle progression through mitogenesis, supporting the autocrine mode of regulation. Finally, we demonstrated that EGFR activation is not required for the autocrine regulation of cell proliferation by ERα.

## Methods

### Chemicals and antibodies

Antibodies for ERα (F10), Ki-67 (H-300), cyclin A (C-19), Cyclin B (H-20), cyclin D1 (H-295), cyclin E (C-19) were obtained from Santa Cruz (Santa Cruz, CA). Blocking peptides for ERα (F10), cyclin A (C-19), cyclin E (C-19) were obtained from Santa Cruz (Santa Cruz, CA). Antibodies for ERK1/2 (137F5), phospho-ERK1/2 (E10), AKT, phospho-AKT/Ser473, and EGFR were obtained from Cell Signaling Technology (Danvers, MA). Secondary antibody conjugated with Alexa Fluor 488 (Molecular Probes, Inc, Eugene, OR) was used for green fluorescent staining, secondary antibody conjugated with Rhodamine Red-X (Jackson ImmunoResearch, West Grove, PA) was used for red fluorescent staining. Heat inactivated FBS and Charcoal-Dextrin stripped FBS (CD-FBS) were obtained from Omega (Tarzana, CA). Estrogen was obtained from Sigma (St. Louis, MO). ICI182780 was obtained from TOCRIS (Ellisville, MO). Gefitinib was obtained from LC Laboratories (Woburn, MA). EGF was obtained from Invitrogen (Carlsbad, CA).

### Cell culture and treatments

DMEM/F12 medium (MediaTech Inc, Herndon, VA) containing 10% heat inactivated FBS and 5 μg/ml insulin was used for routine maintenance of the three ERα-positive breast cancer cell lines, MCF-7, T47D, and ZR75-1 (ATCC). For experimental assays, phenol-red free DMEM/F12 (MediaTech Inc, Herndon, VA) containing 5% CD-FBS was used unless otherwise specified. To arrest cells in G1 phase by ICI182780, cells were seeded in culture dishes for 1–2 days to let the cells reach about 30–40% confluency before replacement with medium containing ICI182780. To evaluate the effect of estrogen stimulation, cells were stimulated by adding E2 either after removal of ICI182780 or in the presence of ICI182780. For estrogen stimulation after removal of ICI182780, cells treated with ICI182780 were washed briefly two times with serum-free phenol red-free DMEM/F12 to remove ICI182780, followed by replacement with medium containing 5 nM estrogen but without ICI182780. The control cells were replaced with medium containing vehicle only. For estrogen stimulation in the presence of ICI182780, cells were treated with 10 nM ICI182780 for various times before adding ten fold of E2 (100 nM) to the cells without removing ICI182780. ICI182780 stock solution was made at 10 mM in DMSO, then serially diluted in DMSO to 10 μM before diluted in culture medium. Estrogen stock solution was made at 10 mM in ethanol, then serially diluted in ethanol to 10 μM before diluted in culture medium. To inhibit the EGFR activation, cells were treated with Gefitinib for 2 hr first; without removing Gefitinib, estrogen or EGF was then added to the cells for stimulation. Gefitinib stock was made in DMSO at 1–50 mM, the stock was diluted in culture medium to make the final concentrations at 1–50 μM.

### Immunofluorescent staining

Cells grown on glass coverslips were briefly washed with ice-cold Dulbecco's PBS and fixed with cold 4% paraformaldehyde containing 0.01% Triton X-100 for 25 min on ice. After washing once briefly with PBS-T (Dulbelcco's PBS containing 0.01% Triton X-100), the cells were treated with 0.05% Triton x-100 in PBS for 10 min for permeabilization. The cells were washed once briefly with PBS-T, followed by blocking in PBS-T containing 5% normal serum and 50 mM NH4Cl for 30 min at room temperature. The cells were then incubated with primary antibodies diluted in PBS-T containing 1% normal serum. Anti-ERα antibody was diluted at 1:50, anti-Ki-67 at 1:100, anti-cyclin D at 1:150, anti-cyclin E at 1:100, anti-cyclin A at 1:100, and anti-cyclin B at 1:100. For the antibodies with blocking peptides available, the antibodies (ERα, cyclin A, cyclin E) were evaluated for its binding specificity by incubation with 5 fold of blocking peptides before applying to cells for primary antibody incubation. After overnight incubation at 4°C, the cells were washed four times with PBS-T, 7 min each time. Then the cells were incubated for 1 hr at room temperature with fluorescent dye-conjugated secondary antibodies (1:200) in PBS-T containing 1% normal serum. The cells were washed 4 times with PBS-T, 7 min each time before mounted on slides with VectoShield with DAPI (Vector Laboratories, Burlingame, CA). Immunofluorescent staining(s) was observed under Olympus BX50 Fluorescence Microscope with Optronics MagnaFire digital camera (Microscope Image Center, UVM).

### Flow cytometry analysis

For analysis of cell cycle distribution, cells were harvested by trypsinization, pelleted by centrifugation, and washed once with Dulbecco's PBS. While vortexing cells, ice-cold 70–75% ethanol was added drop wise to resuspend the cells. The cells were fixed in 70% ethanol for at least 1 hr at 4°C before staining or stored at -20°C freezer. Before staining, 1–3 millions of cells were pelleted, washed once with PBS, followed by resuspension in 1 ml PBS containing 20 μg/ml PI (propidium iodide), 150 μg/ml RNaseA, 10 mM EDTA, and 0.1% Triton X-100. Incubation was carried in the dark at 37°C for 15–30 min. For the analysis of cell cycle distribution of ERα-staining cells, cells were harvested by trypsinization and the cell number counted. The cells were pelleted by centrifugation (2000 rpm, 5 min, 4°C). After resuspension with culture medium, 3 million of cells were transferred into Ependorff tube and pelleted by centrifugation (500 × g, 4°C, 2 min). The cell pellet was resuspended in 1 ml PBS-T containing 1% paraformaldehyde and cells were fixed on ice for 10 min. The cells were pelleted by brief centrifugation, resuspended in PBS containing 0.05% Triton-X100 for permeabilization at room temperature for 5 min. After brief washing, cells were incubated with 5% normal serum in PBS-T (30 min, room temperature) for blocking. Cells were pelleted and resuspended in PBS-T containing anti-ERα (F10, 1:100 dilution) and 0.5% BSA, followed by incubation at 4°C for overnight. The cells were washed 4 times with PBS-T, 7 min each, by centrifugation and resuspension, followed by incubation with the secondary antibody anti-mouse Alexa Fluor 488 (1:300) at room temperature for 40 min. After washing with PBS-T for 4 times, 7 min each, the cells were incubated with 0.5 ml PI-RNaseA solution for 30 min at 37°C. Flow cytometry analysis was performed using the Coulter Epics XL-MCL (VCC core facility, UVM). About 20,000 cells were analyzed for each sample.

### Protein analysis by immuno-blotting

Cells at 70–80% confluence were washed briefly twice with ice-cold D-PBS before scraping on ice with lysis buffer (20 mM Tris-HCl, pH 7.5; 150 mM NaCl; 2.5 mM sodium pyrophosphate; 1 mM sodium β-glycerophosphate; 5 mM NaF, 1 mM Na3VO4, 1 mM phenylmethylsulfonyl fluoride, 1% Triton X-100, and 1 tablet of protease inhibitor mixture (Roche Molecular Biochemicals, Indianapolis, IN) per 40 ml of lysis buffer). Cellular debris was removed by centrifugation (14,000 × *g *for 15 min at 4°C). For SDS-PAGE, total cell lysate containing ~30 μg proteins were separated on 12% SDS-PAGE gel and transferred onto PVDF membranes (Millipore, Bedford, MA). Immunoblotting was carried out followed standard procedures [37,38].

## Results

### Colocalization of ERα and Ki67 in ERα-positive breast cancer cell lines

**Figure 1:**
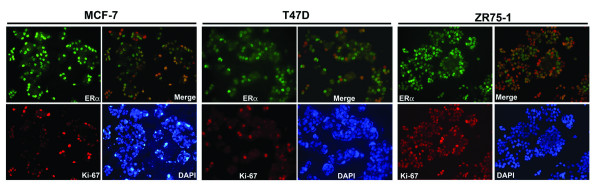
**Colocalization of ERα and Ki-67 in ERα-positive breast cancer cell lines**. MCF-7, T47D, and ZR75-1 cells were co-immunofluorescent stained for ERα (green) and Ki-67 (red). ERα-staining and Ki-67 staining were overlayed to show colocalization (Merge). Nuclei were stained with DAPI. Shown here are photographs of cells grown in phenol-red free DMEM/F12 containing 5% CD-FBS (200× magnification).

ERα is not colocalized with the cell proliferation marker Ki-67 in normal mammary cells, and colocalization of ERα and Ki-67 is rare even in primary breast tumors [1-3,16-18,23]. To study the mechanism of how cell proliferation is mediated by ERα, the ERα-positive breast cancer cell lines were used in our study. In all the three ERα-positive cell lines evaluated in this study, i.e., MCF-7, T47D, and ZR75-1, a large proportion of cells showed duel-staining of ERα and Ki-67 (Fig. 1). The colocalization of ERα with Ki-67 was evaluated in cells under different culture conditions and the duel-staining was observed in all culture conditions, including cell culture in regular DMEM/F12 supplemented with 10% FBS, phenol-red free DMEM/F12 supplemented with charcoal dextrin stripped FBS, with and without insulin, with and without estrogen. While these data don't exclude the possibility of a paracrine regulation of cell proliferation by ERα in these cells, the colocalization of ERα and Ki-67 does support the hypothesis that ERα may mediate cell proliferation in an autocrine mode in these ERα-positive breast cancer cell lines, a mechanism absent in normal mammary cells and most primary breast tumors [1-3,16-18,23,24].

### Deregulated expression of ERα in all phases of cell cycle

**Figure 2:**
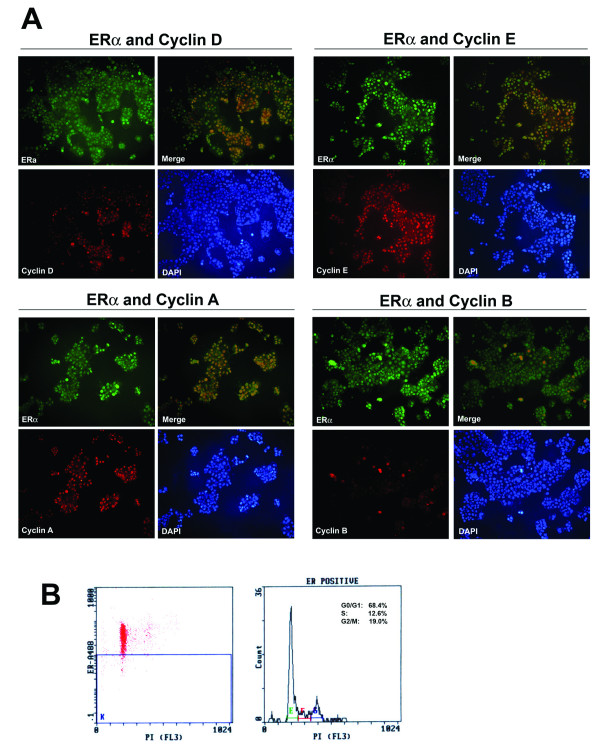
**Deregulated expression of ERα in the cell cycle in MCF-7 cells**. A, co-immunofluorescent staining for ERα (green fluorescence) and the major cyclins (D, E, A, and B) (red fluorescence). Cells were immunofluorescent stained with antibody for ERα and the antibody for one of the cyclins, followed by secondary antibody. Colocalization was shown by overlaying (Merge). Magnification, 200×. B, Flow cytometry analysis for the cell cycle distribution of ERα^high ^cells (cells with high levels of ERα). Cells were stained with antibody for ERα followed by Alexa Fluor 488, and nuclear DNA was stained with propidium iodide (PI).

In normal breast tissue, ERα is expressed in quiescent (G_0_) cells or early G_1 _phase [1,17]. In MCF-7 cells, activation of ERα leads to cyclin D1 and myc expression, indicating the expression of ERα in G1 phase [26]. Ki-67 is a proliferation marker expressed in late G1, S, G2, and M phases of the cell cycle [39]. In these ERα-positive breast cancer cell lines, most Ki-67-staining cells contained high levels of ERα suggesting that ERα might be present throughout the cell cycle progression in these cell lines (Fig. 1). To address this possibility, we evaluated the colocalization of ERα with the cyclins for different phases of cell cycle (cyclin D for G1 phase; cyclin E for late G1 phase; cyclin A for G1/S/G2 phases; cyclin B for M phase) [40-42]. The cyclins for the different phases of cell cycle were found colocalized with a proportion of ERα cells, with the cyclin A showing the largest and cyclin B the least proportion of ERα-staining cells (Fig. 2A). Furthermore, flow cytometry analysis demonstrated that MCF-7 cells with high ERα expression (ERα^high^) were distributed across G0/G1, S, and G2/M phases (Fig. 2B). These data indicate that ERα expression during cell cycle progression is deranged in MCF-7 cells.

### E2 stimulation of ICI182780 treated cells induces expression of ERα and Ki-67

**Figure 3:**
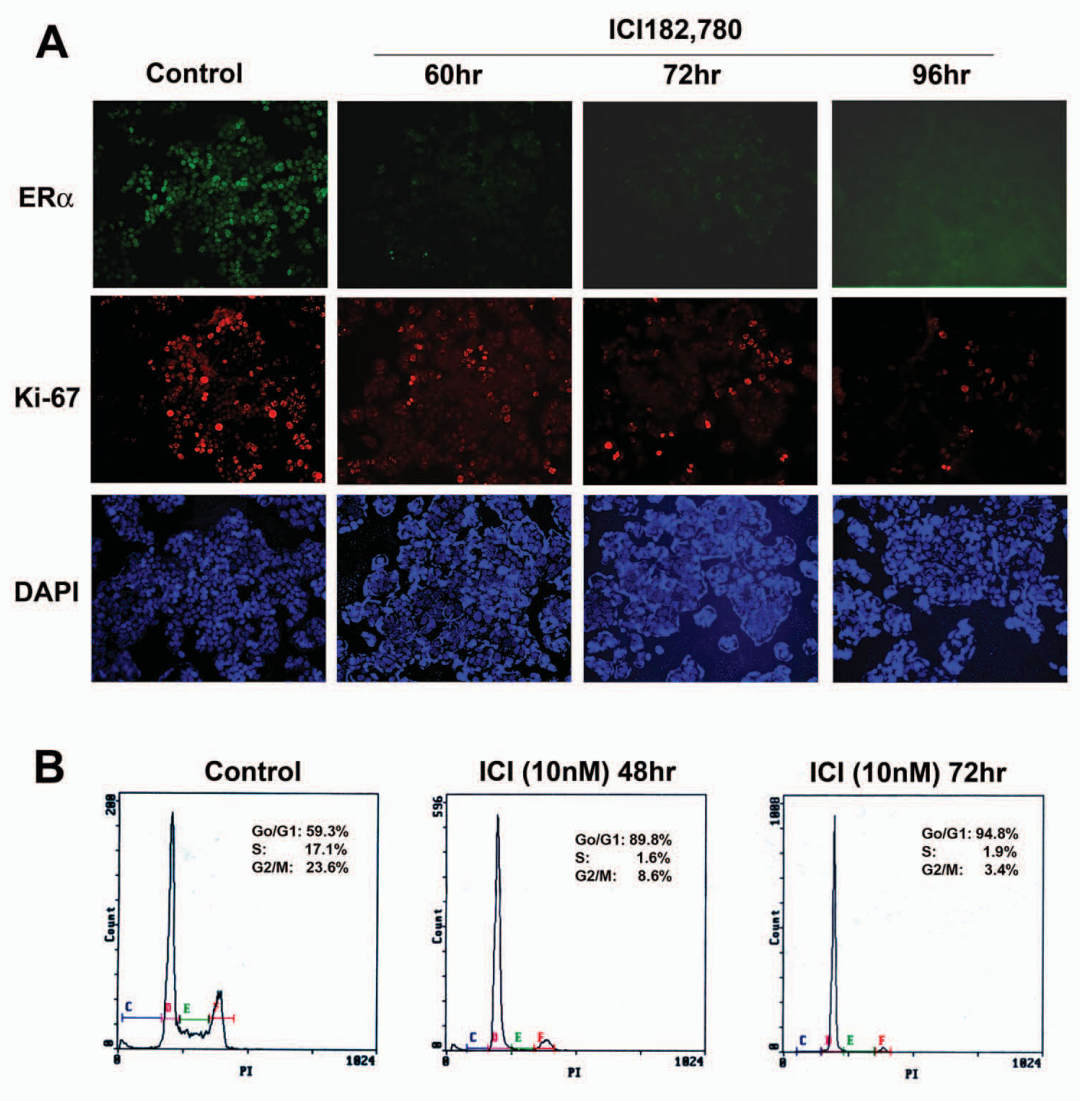
**ICI182780 treatment of MCF-7 cells leads to ERα down-regulation and cell cycle arrest**. A, Down-regulation of ERα by 10 nM ICI182780 treatment for 60–96 hrs. ERα and Ki-67 were evaluated by immunofluorescent staining, nuclei were stained with DAPI. Cells without ICI182780 treatment were used as control. Magnification, 200×. B, flow cytometry analysis of cell cycle distribution. Shown here is the cell cycle distribution of non-treated control cells, and cells treated with 10 nM ICI182780 for 48–72 hr. ICI182780 treatment was carried out in phenol-red free DMEM/F12 containing 5% CD-FBS.

While the distribution of ERα across all phases of cell cycling supports the autocrine mode of action in cell proliferation, it also raises the possibility that the colocalization of ERα with Ki-67 might be a coincidence. To further determine whether ERα can mediate cell proliferation in an autocrine mode, we sought to arrest the cells with the pure ERα antagonist ICI182780 followed by estrogen stimulation. When treated with ICI182780 for 72–96 hrs, no cell was observed with high ERα expression and only a few cells were stained with Ki-67, indicating that both ERα and Ki-67 were down-regulated by ICI182780 (Fig. 3). Consistently, ICI182780 treatment leads to cell cycle arrest at G1 phase (Fig. 3). The inhibitory effect of ICI182780 was observed similarly at concentrations from 10 nM to 50 nM, therefore 10 nM was selected for our later experiments.

**Figure 4:**
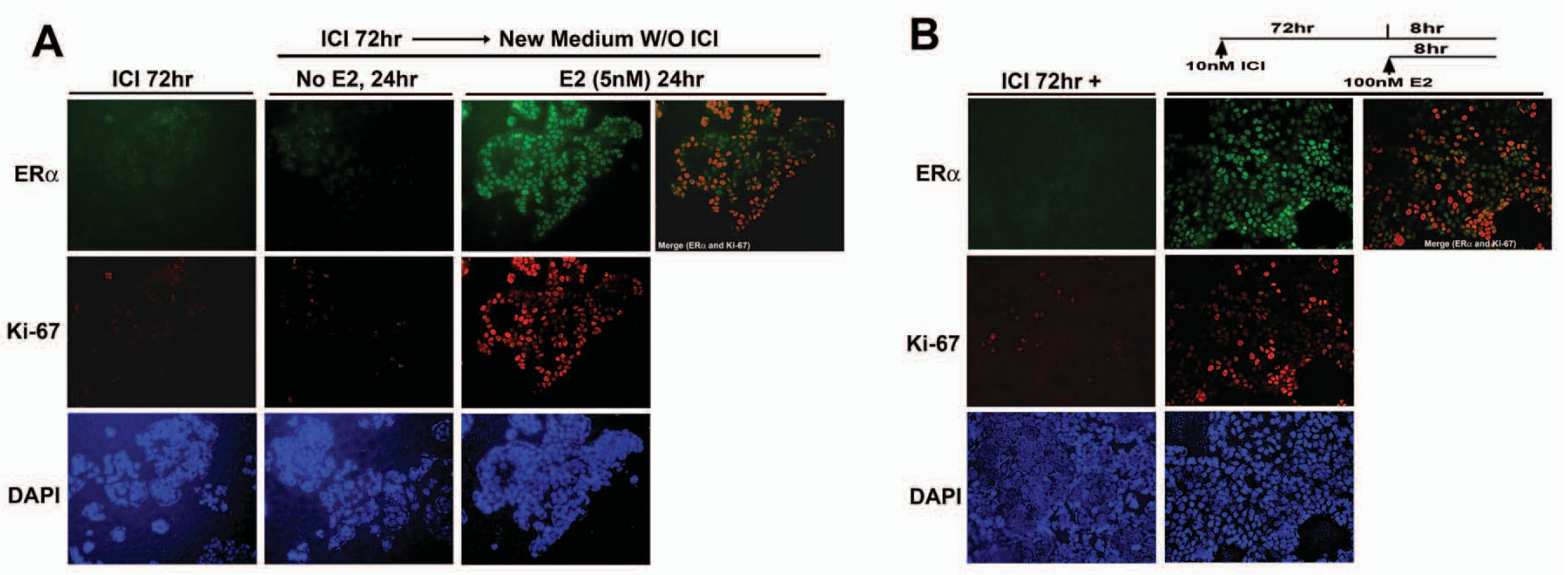
**E2 stimulation of ICI182780 treated cells induced the expression and colocalization of ERα and Ki-67**. A, E2 stimulation of cells released from ICI182780 treatment. MCF-7 cells were treated with ICI182780 for 72–96 hr, then ICI182780 was removed by washing with serum-free medium. After removal of ICI182780, cells were treated by 5 nM E2 for 24 hr; control cells were replaced with medium without E2. Magnification, 200×. B, stimulation of E2 by adding E2 directly to cells under ICI182780 treatment. Cells were treated with 10 nM ICI182780 for 72–96 hr, then 100 nM E2 was added directly to the cells without removing ICI182780 for estrogen stimulation. The expression of ERα and Ki-67 was evaluated by immunofluorescent staining. Overlay of the ERα-staining and Ki-67 staining showed the colocalization (Merge). DAPI was used for nuclear staining. Magnification, 200×.

To evaluate the effect of estrogen stimulation, cells were stimulated by adding E2 either after removal of ICI182780 or in the presence of ICI182780. We first evaluated the effect of estrogen stimulation after removal of ICI182780. MCF-7 cells were treated for ICI182780 for 72–96 hr and the treatment was stopped by washing with fresh serum-free medium to remove ICI182780. The cells were then stimulated with estrogen by adding medium containing 5 nM estrogen but without ICI182780. The control group of cells was replaced with medium without estrogen. In the cells stimulated with estrogen, both ERα and Ki-67 were induced with ERα and Ki-67 colocalized in most cells (Fig. 4A). In the control group of cells without estrogen stimulation, no high level of ERα or Ki-67 was observed although the cells were released from ICI182780 treatment. We also determined the effect of estrogen stimulation by adding estrogen directly to the cells still under ICI182780 treatment (Fig. 4B). The continuous presence of ICI182780 was to help minimize the secondary factors that might promote cell proliferation. Cells were treated with 10 nM ICI182780 for 72–96 hrs before the estrogen was added to the cells for a final concentration of 100 nM. Similar to the result shown in Fig. 4A, excessive estrogen stimulation in the presence of ICI182780 also induced the expression of high levels of ERα and Ki-67 with the two proteins colocalized in most cells (Fig. 4B). Collectively, these data indicate that activation of ERα by estrogen in ICI182780 arrested cells induces the expression of high levels of ERα and Ki-67 and their colocalization, supporting the autocrine mode of ERα action.

### E2 stimulation releases cell cycle arrest by ICI182780 to promote cell cycle progression

**Figure 5:**
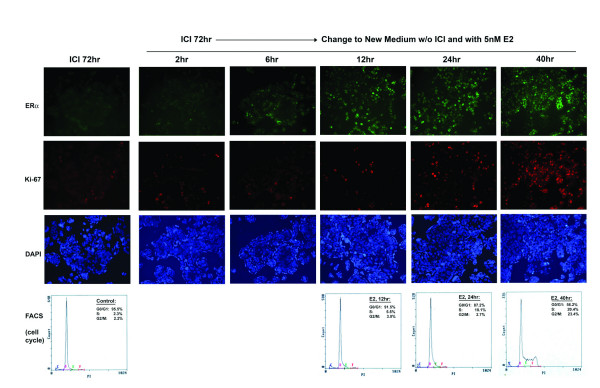
**Time course of E2 induced expression of ERα and Ki-67 and cell cycle progression**. MCF-7 cells were treated with ICI182780 for 72–96 hr and ICI182780 treatment was stopped by washing with serum-free medium. After removal of ICI182780, cells were treated with 5 nM E2 for various lengths of time (from 2 hr to 44 hr). Cells grown on coverslips were immunofluorescent stained for ERα and Ki-67. Nuclei were stained with DAPI. Cells grown on regular culture dishes were collected for PI staining and analyzed by flow cytometry for cell cycle progression. Magnification, 200×.

We next examined the effect of estrogen stimulation on cell cycle progression. As described above, cells were stimulated with estrogen either after release from ICI182780, or estrogen was added at 10 fold higher concentration (100 nM E2) directly to cells without removing ICI182780 (10 nM ICI). Similar results were observed for either stimulation (Fig. 5, and data not shown). In cells stimulated with estrogen after release from ICI182780, most cells were still in the G1 phase until 24 hr after stimulation. At 44 hr after estrogen stimulation, about 45% of the cells were in S or G2/M phases, indicating that activation of ERα promotes the progressing of the cell cycle (Fig. 5). Interestingly, induction of high levels of ERα and Ki-67 appeared much earlier, with high levels of ERα appeared at around 6 hr. It is noteworthy that high levels of ERα appeared earlier than Ki-67, supporting that the activation of ERα is mediating the expression of Ki-67 and the cell cycle progression. These data indicate that the colocalization of ERα and Ki-67 is not a coincidence and support the hypothesis that activation of ERα can promote cell proliferation by autocrine regulation.

### EGFR signaling is not required for the autocrine regulation of cell proliferation by ERα

**Figure 6:**
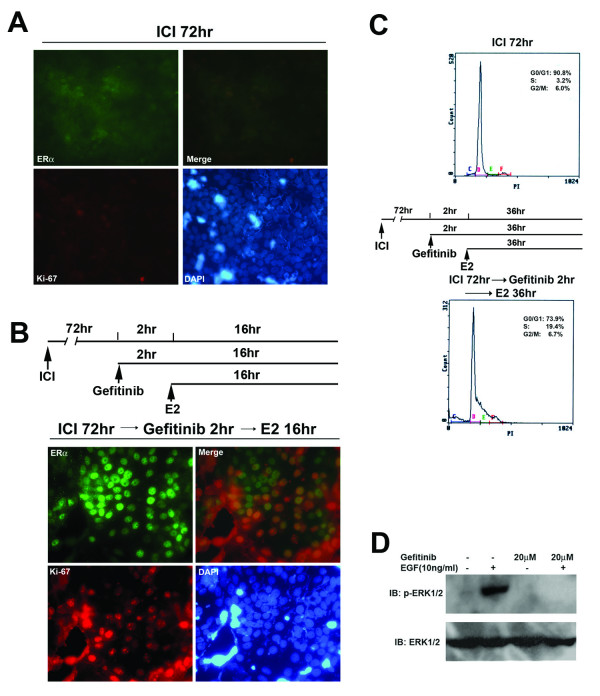
**EGFR signaling is not required for the autocrine regulation of cell proliferation by ERα**. A, MCF-7 cells treated with 10 nM ICI182780 for 72–96 hr, showing the down-regulation of ERα and Ki-67 by ICI182780 treatment. In A and B, ERα and Ki-67 were evaluated by immunofluorescent staining, nuclei were stained with DAPI. ERα-staining and Ki-67 staining were overlayed to show colocalization (Merge). Magnification, 400×. B, EGFR inhibitor Gefitinib does not inhibit E2 induced ERα and Ki-67 expression. MCF-7 cells were stimulated with 100 nM E2 in the presence of 10 nM ICI182780 and 20 μM EGFR inhibitor Gefitinib. MCF-7 cells were treated first with 10 nM ICI182780 for 72–96 hr, followed by adding the EGFR inhibitor Gefitinib to a final concentration of 20 μM for 2 hr, before adding estrogen to a final concentration of 100 nM for 16 hr. C, flow cytometry analysis of cell cycle progression, showing E2 stimulated cell cycle progression is not inhibited by Gefitinib. Upper panel, MCF-7 cells treated with ICI182780 for 72 hr. The cells were arrested at G1 phase. Lower panel, cell cycle progression was stimulated by 100 nM E2 in the presence of 10 nM ICI182780 and 20 μM EGFR inhibitor Gefitinib. MCF-7 cells were treated first with 10 nM ICI182780 for 72 hr, followed by treatment with the Gefitinib (20 μM) for 2 hr, before stimulation with 100 nM E2 for 36 hr. D, immunoblotting of phosphorylated ERK1/2, showing the inhibition of EGFR signaling by 20 μM Gefitinib. MCF-7 cells were serum starved for 24 hr, followed by treatment with 20 μM Gefitinib for 2 hr, before EGF stimulation for 5 min. The control group of cells was not treated with Gefitinib. Phosphorylation of ERK1/2 was used to assess the activation of the EGFR signaling.

In the paracrine regulation of cell proliferation by ERα, EGFR signaling is required [20]. Therefore we evaluated whether EGFR signaling is required for the autocrine regulation of cell proliferation by ERα in MCF-7 cells. EGFR inhibitor Gefitinib was used to block EGFR signaling, 20 μM of Gefitinib was chosen based on our preliminary experiments using a wide range concentrations of Gefitinib (1 μM – 50 μM) (Fig. 6D, and data not shown). We first evaluated whether Gefitinib treatment inhibits E2 stimulation induced expression and colocalization of ERα and Ki-67. MCF-7 cells were treated first with 10 nM ICI182780 for 72–96 hr, followed by adding the EGFR inhibitor Gefitinib to a final concentration of 20 μM for 2 hr, then by adding estrogen to a final concentration of 100 nM. In the presence of ICI182780 and Gefitinib, estrogen stimulation still induced the expression and colocalization of ERα and Ki-67 (Fig. 6B). Conceivably, Gefitinib treatment does not inhibit estrogen stimulation induced cell cycle progression (Fig. 6C lower panel). In the control groups of MCF-7 cells treated with ICI182780 for 72–96 hr, ERα and Ki-67 were down-regulated and the cells remained at G1 phase (Fig. 6A, C upper panel). In the growth curve studies and the EGF stimulation experiments, Gefitinib at 20 μM is sufficient to block EGFR signaling but not toxic to the cells (Fig. 6D, and data not shown). From these data, we concluded that EGFR signaling is not required for the autocrine action of ERα in mediating cell proliferation in MCF-7 cells.

## Discussion

ERα is the primary estrogen receptor mediating the effects of estrogen in normal mammary epithelial cells [1-3,5-9,43]. In breast cancer, abnormal expression of ERα is found in about 70% of primary tumors [10,13]. Despite the significant role of ERα, the mechanism of how ERα mediates cell proliferation is not fully understood. The dissociation of ERα with cell proliferation markers such as Ki-67 in the normal mammary gland leads to the paracrine model of ERα-mediated cell proliferation. While colocalization of ERα and Ki-67 is rare in most primary breast cancers, there do exist some cancer cells in some primary breast tumors showing colocalization of ERα and Ki-67 suggesting the possibility of autocrine regulation. In this paper using the ERα-positive breast cancer lines, we presented data showing that most Ki-67 staining cells expressed high levels of ERα and that cells with high levels of ERα are distributed across different phases of cell cycle. We further demonstrated that activation of ERα in ICI182780 arrested cells can induce the expression and colocalization of high levels of ERα and Ki-67, and more importantly, the progressing of cell cycles from G1 phase to the G2/M phases. Collectively, our data support the hypothesis that ERα can mediate cell proliferation in an autocrine mode in these ERα-positive breast cancer cell lines.

Whether ERα mediates cell proliferation by autocrine and/or paracrine regulation is related to whether ERα-positive cells can proliferate or not. Inferred from the paracrine model is that ERα-positive cells are a separate sub-population of cells that don't proliferate, or even further that ERα-positive cells cannot proliferate because ERα inhibits the proliferation of ERα-positive cells [1,3,44,45]. Although ERα is known to promote cell proliferation in ERα-positive breast cancer cell lines such as MCF-7, exogenous overexpression of ERα in some cell lines such as MDA-MB-231 and MCF-10A does inhibit cell proliferation [46-50]. The mechanism of how ERα mediates cell proliferation may have clinical implication for breast cancer etiology and therapy. Malignant transformation is a multistep process that involves different combination of genes at different stage of malignancy [51]. Distinct sets of ERα-target genes might be involved for the autocrine versus paracrine regulation and it will be interesting to examine whether the resistance to endocrine-related therapy is correlated with the mode of ERα action.

All the three ERα-positive cell lines used in our study showed colocalization of ERα and Ki-67. Since the number of established ERα-positive breast cancer cell lines is much less than expected from the expression frequency of ERα in primary breast tumors, we speculated that ERα-positive cell lines could only be established from cancer cells with autocrine regulation in the primary tumors [13,52]. In that case, what is known about the breast cancer from the studies using the ERα-positive breast cancer cell lines may not apply to all the ERα-positive primary breast tumors. It needs to point out that although our data support the autocrine mode of ERα action, it remains to determine whether both autocrine and paracrine modes of action by ERα are involved in these ERα-positive breast cancer cell lines. ERα is expressed in both the nucleus and cell surface in MCF-7 cells [53,54]. Based on the levels of membrane ERα, MCF-7 cells can be separated into two sub-populations, mERα^high ^and mERα^low ^[55,56]. The levels of nuclear ERα also showed heterogeneity in MCF-7 cells. It is not clear whether the heterogeneity of nuclear ERα levels is caused by the differential expression of ERα during cell cycle progression or by the heterogeneous sub-populations of cells. As demonstrated in ICI182780 treated cells, even extremely low or basal level of ERα can mediate some of the cellular response to estrogen. To determine whether paracrine regulation is involved or not, a subline of MCF-7 with the ERα being completely shut off, i.e., eliminating the low basal level of ERα, will be needed to test their proliferation in response to estrogen when mixed with normal MCF-7 cells.

Together with the systemic hormones, growth factors are involved in mammary cell proliferation. EGFR signaling is essential for ERα-mediated cell proliferation in the normal mammary glands [20]. In MCF-7 cells, EGFR is involved in the non-genomic action of ERα and blocking of some EGFR activated pathways could inhibit ERα-mediated cell proliferation [33-36]. Our data, however, indicate that EGFR signaling is not required for the autocrine regulation of cell proliferation by ERα.

## Conclusion

ERα can mediate estrogen-induced cell proliferation in an autocrine mode in ERα-positive breast cancer cell lines. All of the three ERα-positive cell lines used in our study showed colocalization of ERα and Ki-67, indicating that these cell lines might be originated from primary tumor cells with autocrine regulation. Considering that the number of established ERα-positive breast cancer cell lines is much less than expected from the percentage of ERα-positive primary breast tumors, we speculated that ERα-positive cell lines could only be established from cancer cells with autocrine regulation in the primary tumors. In that case, what is learnt about the breast cancer from the studies using the ERα-positive breast cancer cell lines may not apply to all the ERα-positive primary breast tumors.

## Competing interests

The authors declare that they have no competing interests.

## Authors' contributions

HT carried out most of the co-localization and cycle assays. YZ carried out the studies using EGFR inhibitor. ZZP was the principal investigator, contributed to the study conception, design and conduct, and writing of the manuscript. All authors read and approved the final manuscript.

## Pre-publication history

The pre-publication history for this paper can be accessed here:

http://www.biomedcentral.com/1471-2407/9/31/prepub
